# How to maintain and transport equine adipose tissue for isolating mesenchymal stem cells?

**DOI:** 10.1186/s12917-022-03379-1

**Published:** 2022-07-21

**Authors:** Faezeh Rezaei, Samira Khasaf, Samaneh Ghasemi, Abbas Parham, Pezhman Mirshokraei

**Affiliations:** 1 Department of Clinical Sciences, School of Veterinary Medicine, Ferdowsi University of Mashhad, Mashhad, Iran; 2 Department of Basic Sciences, Faculty of Veterinary Medicine, Ferdowsi University of Mashhad, Mashhad, Iran; 3 Stem Cell Biology and Regenerative Medicine Research Group, Institute of Biotechnology, Ferdowsi University of Mashhad, Mashhad, Iran; 4 Center of Excellence in Ruminant Abortion and Neonatal Mortality, School of Veterinary Medicine, Ferdowsi University of Mashhad, Mashhad, Iran

**Keywords:** Equine, Mesenchymal stem cell, Adipose tissue

## Abstract

**Background:**

Adipose tissue (AT) is one of the most important mesenchymal stem cell (MSC) sources because of its high quantities, availability and ease of collection. After being collected samples, they should be transported to a laboratory for stem cell (SC) isolation, culture and expansion for future clinical application. Usually, laboratories are distant from animal husbandry centers; therefore, it is necessary to provide suitable conditions for adipose tissue transportation, such that adipose-derived MSCs are minimally affected. In the current study, the impact of tissue maintenance under different conditions on MSCs derived from these tissues was evaluated. We aimed at finding suitable and practical transportation methods in which ASCs go through the slightest changes.

**Results:**

In the current study, after being collected, equine AT was randomized into eight groups: four samples were maintained in stem cell culture media at 25 ^ο^C and 4 ^ο^C for 6 and 12 hrs. as transportation via SC media groups. Three samples were frozen at three different temperatures (− 20, − 75 and − 196 ^ο^C) as cryopreserved groups; these samples were defrosted 1 week after cryopreservation. Fresh and unfrozen AT was evaluated as a control group. The tissue samples were then initiated into enzymatic digestion, isolation and the culturing of SCs. Cells at passage three were used to evaluate the ability to form colonies, proliferation rate, plotting of the cell growth curve, and viability rate. All experiments were performed in triplicate. Stem cell isolation was successful in all groups, although purification of SCs from the first series of cryopreservation at − 196 ^ο^C and two series of − 20 ^ο^C was unsuccessful. There was no significant difference between the surface area of colonies in all groups except for − 20 ^ο^C. The growth rate of transportation via stem cell media at 25 ^ο^C for 6 hrs. was similar to that of the control group. MTT analysis revealed a significant difference between 25 ^ο^C 12 hrs. Group and other experimental groups except for control, 4 ^ο^C 12 hrs. and − 196 ^ο^C group.

**Conclusion:**

Data have shown freezing at − 75 ^ο^C, transportation via stem cell media at 4 ^ο^C for 12 hrs. and 25 ^ο^C for 6 hrs. are acceptable tissue preservation and transportation methods due to minor effects on MSCs features.

**Supplementary Information:**

The online version contains supplementary material available at 10.1186/s12917-022-03379-1.

## Background

Over the last few years, stem cells have come to the researchers’ attention [1–3]. Mesenchymal stem cells are regarded as one of the most favorable cell types for regenerative medicine because of their properties and therapeutic prospects, such as differentiation potential into the various mesenchymal lineage, anti-inflammatory, modulation of immune responses, angiogenic characteristics and low immunogenicity, which makes them ideal for allogenic cell-based therapies [4–9]. Currently, MSCs are utilized to address musculoskeletal disorders in veterinary medicine [10–15]. Moreover, the cell-based treatment seems to be a promising future therapy for a wide variety of different disorders like autoimmune, neurological, cardiovascular, ophthalmological and hepatic disorders and skin wound healing [16–21]. Mesenchymal stem cells originate from various sources, mainly bone marrow-derived MSCs and adipose-derived stromal/stem cells (ASC) have been extensively utilized to treat different animal diseases [1, 22]. Adipose tissue constitutes a good and available source of MSCs in equine medicine [11, 23, 24]. In comparison with the other sources of SCs, AT can be obtained easier, which is the main benefit of this tissue [23, 25]. Furthermore, the frequency of ASCs in AT is greater than the frequency of bone marrow-derived stem cells [26].

To use autologous ASCs for clinical application and treatment of ligament and tendon disorders and joint diseases in horses, the fat sample is extracted and sent to the laboratory for stem cell isolation and expansion. Processed samples are returned to a veterinarian for therapeutic application [27]. Hence utilization of ASCs for the clinical application requires finding a suitable method to maintain the collected tissue, as well as providing appropriate conditions for tissue transportation between animal farms, laboratories, animal medical centers and even between cities.

Commercial tissue transportation is affected by several factors, such as the distance between farms and laboratories, the way of shipment, the amount of transported tissue and the transportation temperature. This study aimed to provide appropriate and feasible conditions for tissue maintenance that ASCs undergo the slightest changes and cells isolated from preserved tissues can be used in future clinical practice. The current study evaluated the effect of temperature and time on ASCs proliferation and viability. The study design was based on the possibility of implementing these methods in the field. Nevertheless, more evaluations and tests, including cell stability validation, chromosomal stability and microbiological tests should be performed in order to utilize isolated cells from transported tissue in animals.

## Results

**Fig. 1:**
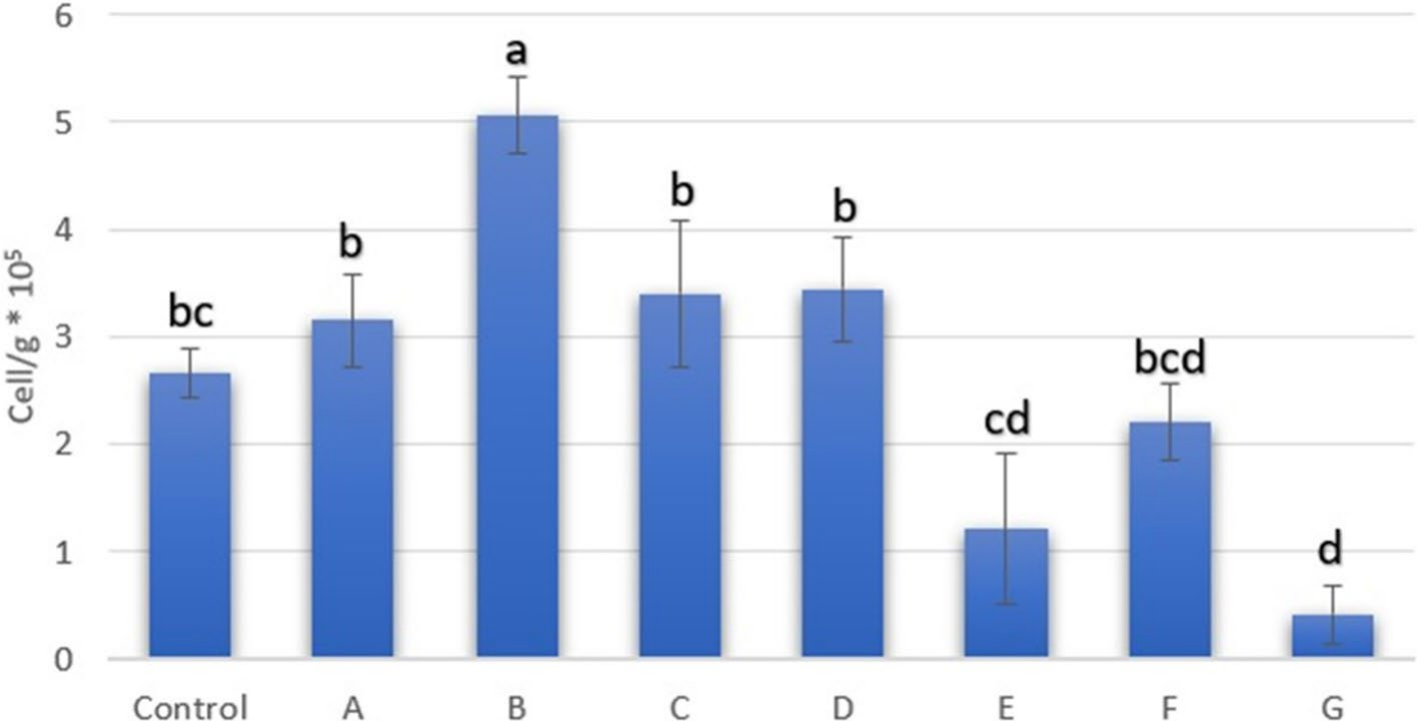
The number of nucleated cells isolated from digested adipose tissue (cell/g × 10^5^); Different superscripts differ significantly (*p* < 0.05)

After mechanical dissociation and enzymatic digestion, adipose-derived nucleated cells were quantified. The cell number is presented in Fig. 1. We isolated SCs from all groups (control, 4 ^ο^C 6 hrs. (A), 25 ^ο^C 6 hrs. (B), 4 ^ο^C 12 hrs. (C), 25 ^ο^C 12 hrs. (D) and − 75 ^ο^C (F)) except for the first series of cryopreservation at -196^ο^C (G) and the first and second series of cryopreservation at − 20 ^ο^C (E).

### Morphological analysis

**Fig. 2:**
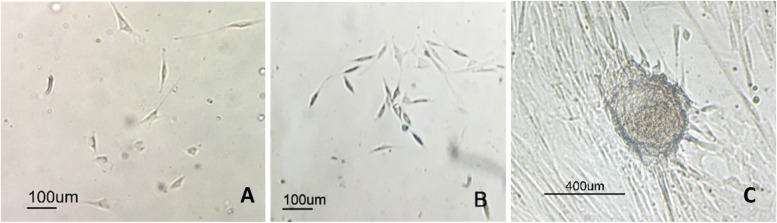
Morphologies of passage 0 ASCs. **A** single adherent cells at day three after seeding (control group). **B** Heterogeneous adherent cells on day five after seeding (group F). **C** On day seven after seeding, nodular aggregation of cells with 90% confluence (control group)

Adherent cells were observed at specific times. On day three after seeding, sporadic adherent cells were observed (Fig. 2-A). On the days four to five after seeding, the number of adherent cells increased and cells varied in morphology (round cells, cells with short cytoplasmic appendages and spindle-shaped cells) (Fig. 2-B). In the cryopreserved groups, cells formed colonies. About 7–12 days after seeding, cells achieved about 70% confluency; at this time, most cells had a fibroblast-like shape (Fig. 2-C).

### Colony-forming assay

**Fig. 3:**
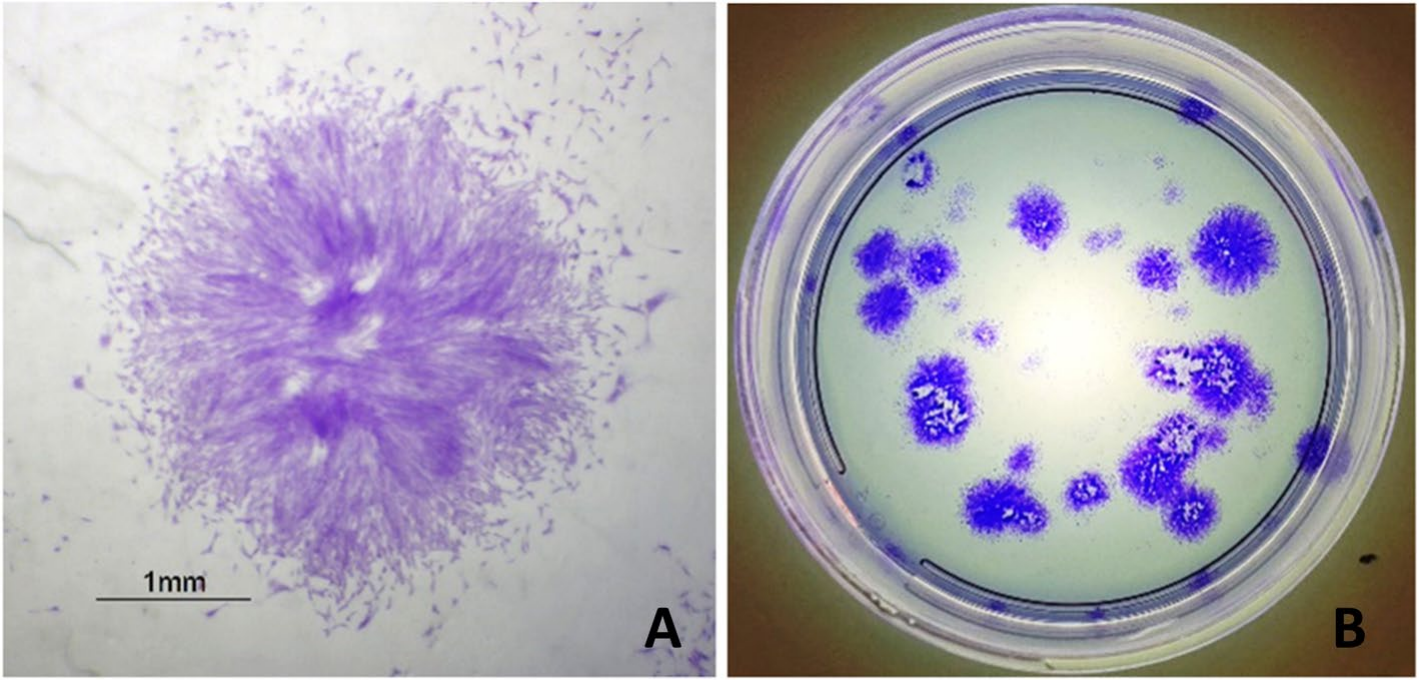
**A** Microscopic illustration of a colony from group C. **B** Macroscopic illustration of colonies formed ten days after seeding from group B

Stem cell colonies are illustrated in Fig. 3. The statistical analysis of the number of colonies and their surface areas is shown in Table 1. In terms of the number of colonies, there was no significant difference. The control group had the highest number of colonies at 52.77 ± 15.32 colonies/100 seeded cells, followed by group G at 44.16 ± 39.83 colonies/100 seeded cells. Group E stood last at 1.66 colonies/100 seeded cells. Analysis of the surface area revealed a significant difference between the group E and the other seven experimental groups. The colony surface area of groups C and A were approximately similar at 29.91 ± 15.37 × 10^4^ pixels and 29.78 ± 13.71 × 10^4^ pixels.

**Table 1:** Colony-forming assay results

Experimental group	Petri dish number	Colonies number/35 mm Petri dish	Surface area (×10^4^) (pixel)
		Mean ± SEM	
Control	9	52.77 ± 15.32	16.62 ± 8.74 ^a^
A	9	32.33 ± 7	29.78 ± 13.71 ^a^
B	9	34.77 ± 6	25.17 ± 14.09 ^a^
C	9	21.22 ± 5.28	29.91 ± 15.37 ^a^
D	9	23.11 ± 9.79	18.03 ± 14.43 ^a^
E	3	1.66	2.15 ^b^
F	9	33.88 ± 16.45	12.61 ± 8.97 ^ac^
G	6	44.16 ± 39.83	3.62 ± 1.26 ^bc^

Different superscripts differ significantly (*p* < 0.05)

### Determining cell proliferation

**Fig. 4:**
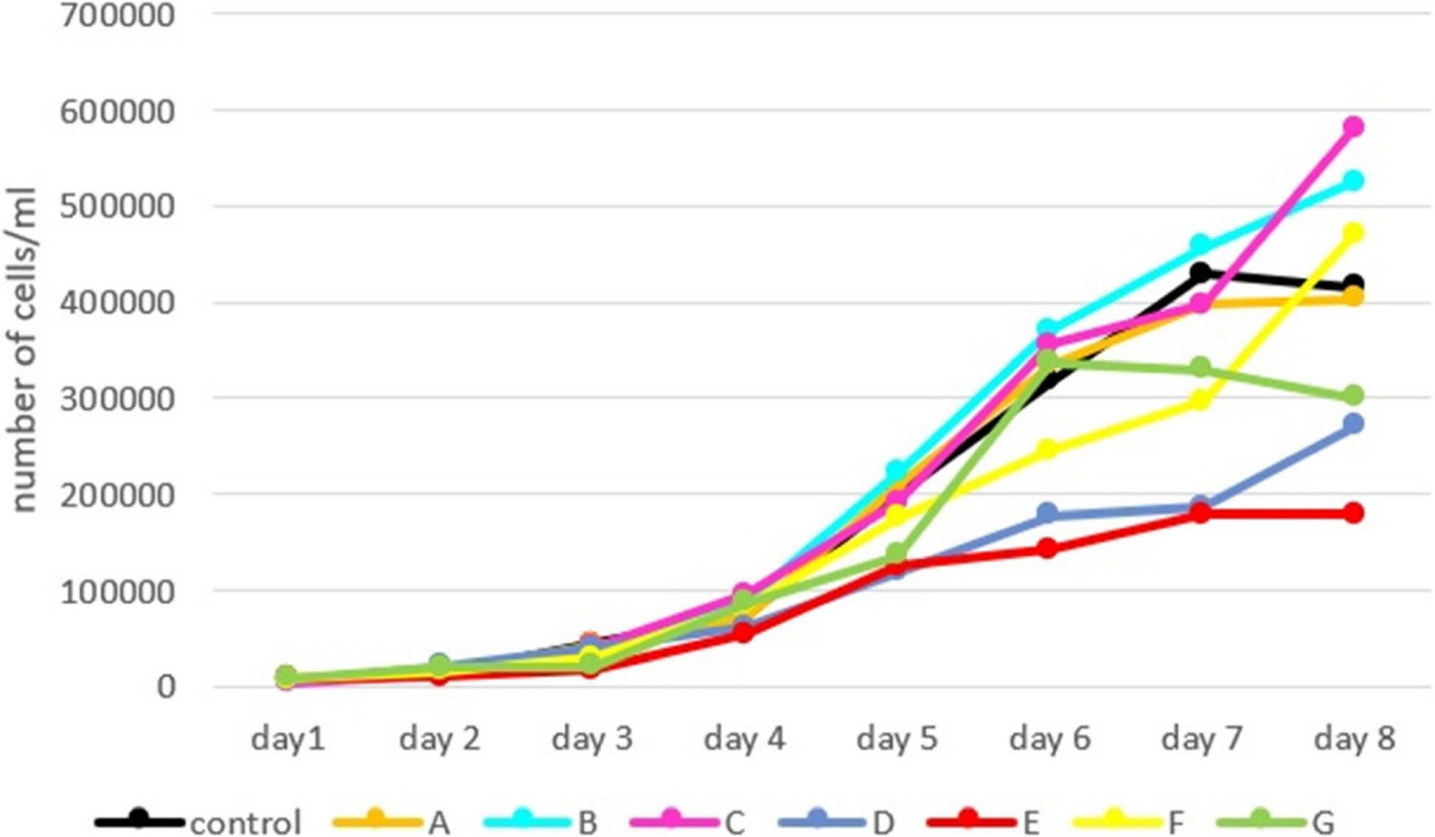
Growth curve of third passaged ASCs, almost all groups follow the same growth pattern. The growth curve includes a latent phase (about day one to two), a logarithmic phase (about days three to six) and a stationary or plateau phase (about days seven to eight). There was a significant difference between the groups B and D, and between B and E, on day seven. There was also a significant difference between C and D

**Fig. 5:**
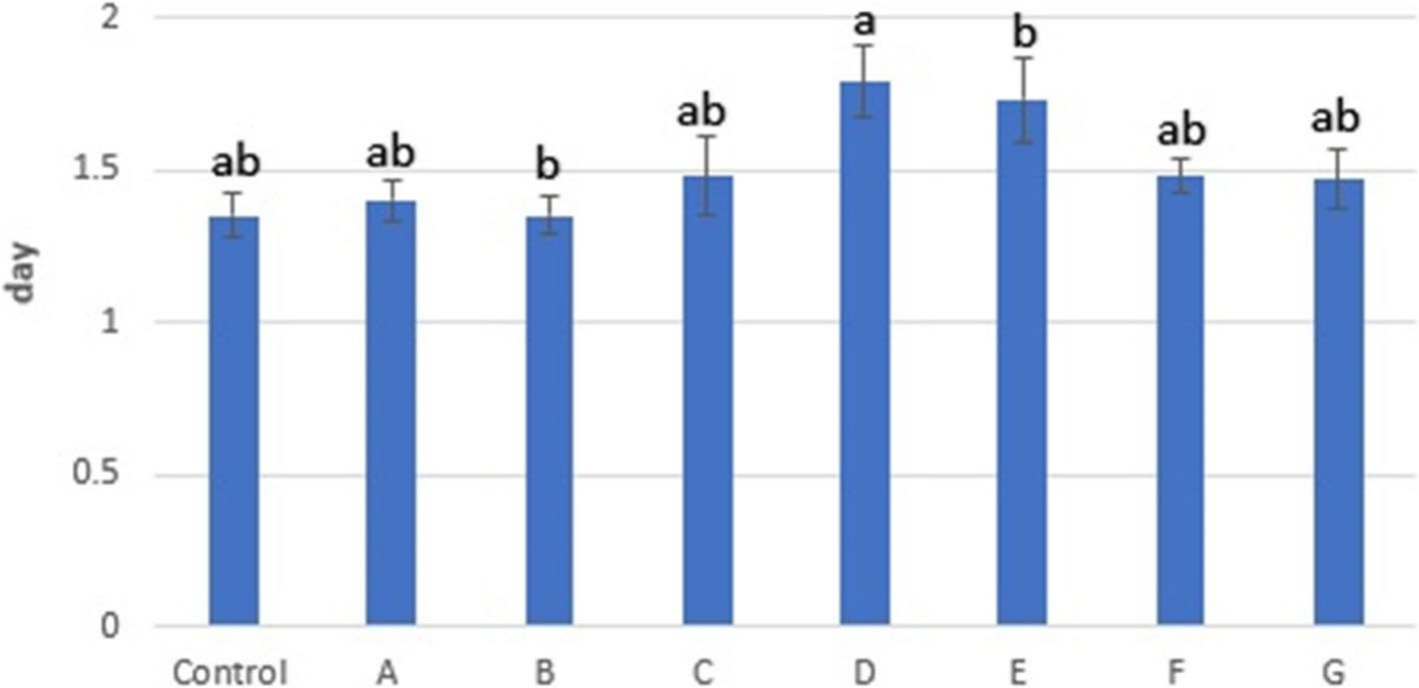
Doubling time results, Different superscripts differ significantly (*p* < 0.05)

After cell numbers were recorded for 8 days, the growth curve was plotted using these data; the growth curve is illustrated in Fig. 4. Statistical analysis revealed no significant difference between the number of cells on different days except for day seven. Based on the growth curve, the stationary phase of the control group was considered day seven, so doubling time (DT) was calculated for all groups at this time. Statistical analyses of DT are presented in Fig. 5. These analyses showed that there was a significant difference between the group D and the other experimental groups, except for the E. Our analyses showed that the Control and group B had the highest growth rate at ≈ 1.35 ± 0.07 days, whereas D had the lowest at 1.79 ± 0.12.

### MTT assay

**Fig. 6:**
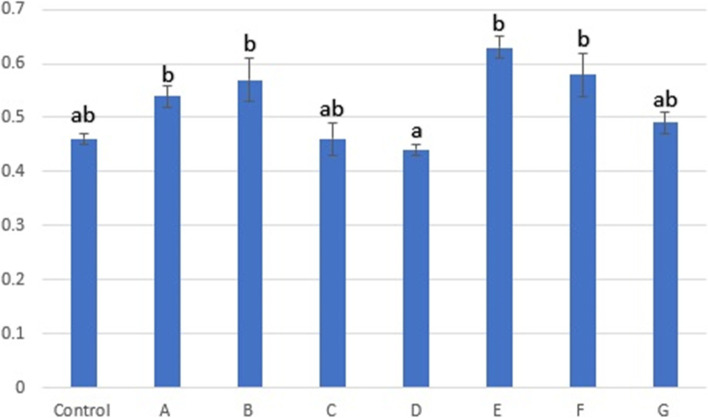
Spectrophotometrically absorbance 560 nm, Different superscripts differ significantly (*p* < 0.05)

This method is a colorimetric assay stand for cellular metabolic activity and proliferation rate. As shown in Fig. 6, there was a significant difference between the mean absorbance at 560 nm (AB_560_) of group D and the other experimental groups except for the control, C and G. Based on AB_560_, the highest absorbance was recorded for group E at 0.63 ± 0.02 which means group E had the highest proliferation rate or cell metabolism. In contrast, the absorbance of group D stood last at 0.44 ± 0.01.

## Discussion

The first step of SC clinical application is appropriate sampling and sample transportation. Almost all farms are distant from laboratories, so there is a need for establishing a method to transfer samples. Different factors affect transportation, such as the distance between farms and labs, the transportation route, such as by car or airplane, the box in which tissue is transported, the volume of transported material, the transportation time and temperature, etc. In the current study, the impacts of the latter two factors are evaluated. It is also noteworthy that tissue samples were excised through a surgery operation rather than liposuction, so the cryoprotectant effect was not studied in the cryopreserved groups.

In the present study, transportation of fat samples at room temperature (25 ^ο^C) or by using a refrigerator (4 ^ο^C), freezer (− 20 ^ο^C), dry ice (− 75 ^ο^C), or liquid nitrogen (− 196 ^ο^C) was assessed. Note that it is feasible to implement these methods on farms.

Heterogeneous morphology of adherent cells was seen four to 5 days after primary seeding. Vidal et al. reported a heterogeneous appearance [28]. Grzesiak et al. described the diversity of cells as being due to different cell population presence, including MSCs, preadipocytes, fibroblasts, smooth muscle and endothelium cells [23].

Arnhold et al. claimed that the CFU assay is a well-founded assay to measure MSCs’ stemness and this method confirms whether a cell population is appropriate for clinical application [29]. The CFU number in the present study was 1.66–52.77 colonies/100 seeded cells. After the control group, the highest number of colonies was recorded in G, while E had the lowest number of colonies. Vidal et al. performed the CFU assay for zero, second and fourth passage ASCs and the number of colonies was reported about 15–43 [28]. In a study by Alipour et al., the number of colonies for third passage equine ASCs was reported 5.33–5.37% [30]. The results of these two studies are consistent with the current study results. However, in a survey by Meirelles et al., the CFU assay was performed for the equine ASCs obtained from stromal vascular fraction; they reported the number of colonies per 100 and 1000 seeded cells were 1–7 and 13–52, respectively. Meirelles and colleagues stated that there is a significant difference between different biological samples [31]. Therefore, the difference in the number of colonies between the current study and the study by Meirelles et al. can be explained. Suga et al. determined the colony area of human pluripotent SCs and the number of fluorescently stained nuclei. They reported a linear relationship between the area of colonies and the cell number. They also claimed that determining the colony areas is a good method to discover cells and colonies’ growth rate, whereas counting cells during passage leads to cell wasting and damage [32]. Orozco-Fuentes et al. showed that the number of cells with smaller nuclei increases when a colony grows and intracellular distance declines [33]. Consequently, colony area analysis reveals the cell proliferation rate. In the current study, cryopreserved groups had smaller colony surface areas in comparison with the control group, while the other four groups’ surface areas were larger.

According to the statistical analysis of the present study, DT was 1.35–1.79 days. In a study by Pall et al. on the first passage equine MSCs, DT was reported at 4.1 ± 0.19. Alipour et al. reported a DT 40–46 hrs. for P_3_ equine MSCs [4, 30]. Vidal et al. in a study on equine ASCs reported that DT for the third passage cells was about 2.4 days [28]. Kim et al. reported different growth rates between cells of various passages [34]. Alipour et al. stated that different culture conditions affect DT [30].

Evaluation of the growth curve showed that the lag phase was short. The logarithmic phase started on day three and continued for about 4 days. It seems that the growth rate of the control, A, E and G groups had declined and the stationary phase of cells started on day seven, but for the rest of the experimental groups, the stationary phase was not seen after 8 days. These results were consistent with the results from the study by Alipour et al. [30].

MTT assay was used to evaluate the cell metabolism. Ghasemi et al. reported that the MTT assay depends on many factors and shows the rate of cell proliferation and metabolism [35]. In the current study, the highest absorbance was recorded in the group E, followed by F. Among groups transported via stem cell media, B had the largest absorbance and A ranked second. Group D had the lowest absorbance. Comparing these data with DT and colony surface area results, which were used to measure cell proliferation rate, showed that although group E had the lowest proliferation rate, it had the highest metabolic activity. In contrast group D have the lowest metabolic activity and low proliferation rate. Consequently, none of these methods are suggested for tissue transportation.

Roato et al., after collecting human AT using liposuction and freezing lipoaspirates at − 20 ^ο^C, − 80 ^ο^C and − 196 ^ο^C, evaluated MSCs viability by MTT assay. They reported that the MSCs viability of cryopreserved samples at − 80 ^ο^C and − 196 ^ο^C was greater than cryopreserved samples at − 20 ^ο^C. Their histological analysis showed that the tissue structures were well preserved. There was no proof of tissue degeneration or necrosis in cryopreserved samples at − 80 ^ο^C and − 196 ^ο^C, comparable to the fresh AT. The analysis of cell mortality after enzymatic digestion revealed that the high number of dead cells in the cryopreserved group had a significant difference with low quantities of dead cells in fresh AT. They showed the protective effect of AT on MSCs-derived tissue in cryopreserved samples [36]. Moscatello et al., after collecting human adipose tissue through liposuction, the lipoaspirates were frozen at − 20 ^ο^C or fat was resuspended in MCDB 201 medium and cryopreserved with or without cryoprotectant at − 80 ^ο^C using a control rate freezing method (1 ^ο^C/min), following samples were preserved at − 196 ^ο^C. Cryopreserved samples were thawed and cultured in plastic cell culture flasks to determine stromal vascular cell viability after cryopreservation. They reported that there were no viable stromal vascular cells in the groups that were frozen without adding cryoprotectant, while they managed to isolate stromal vascular cells in the groups that were frozen with cryoprotectant. They conclude by using a cryoprotectant, utilizing a control-rate freezing method and storage at − 196 ^ο^C provide an appropriate condition that maintains ASCs viability [37]. Lie et al. preserved AT from liposuction procedure at − 20 and − 80 ^ο^C with or without a cryoprotective agent before thawing and assessing the existence of ASCs through seeding stromal vascular fraction. They reported that no ASCs were isolated from frozen samples without cryoprotectant. In contrast, they isolated ASCs from samples with cryoprotectant [38]. Although no cryoprotectant was added in the current study, the isolation of ASCs from cryopreserved ATs was successful. As the cryopreserved samples were collected through a surgical operation rather than liposuction, the ASCs might be protected by adipose tissue.

In the current study, group E had the lowest number of nucleated cells after enzymatic digestion. Group F had higher nucleated cells than G. Li et al. described that intracellular ice formation and osmotic stress following cryopreservation caused cell damage [39]. Wang et al. reported that tissue freezing at − 20 ^ο^C caused ASCs degeneration and apoptosis [40]. Wolter et al. explained that cells had metabolic activity at − 20 ^ο^C. There was no cellular activity at temperatures below − 130 ^ο^C, but freezing cells below − 130 ^ο^C is harmful. They reported that − 80 ^ο^C could be a suitable cryopreservation temperature [41]. These studies are consistent with our current cryopreservation results.

## Conclusion

The current study results have shown that freezing at − 20 ^ο^C is not a suitable method for AT cryopreservation due to poor SCs isolation. The difference between the results of the − 75 ^ο^C and − 196 ^ο^C groups was not significant, although cryopreservation at − 75 ^ο^C is more efficient than at-196 ^ο^C; As in − 75 ^ο^C group SCs isolation was successful in all three series of sampling, while that for − 196 ^ο^C was only in 2 series. Therefore, cryopreservation at − 75 ^ο^C is the best method for adipose tissue transportation among cryopreserved groups.

Comparing other groups revealed that maintaining AT at room temperature for 12 hrs. is not an effective method for tissue transferring. Tissue maintenance at 4 ^ο^C for 6 and 12 hrs. and 25 ^ο^C for 6 hrs. Are acceptable methods due to having minor effects on ASCs.

## Methods

**Fig. 7:**
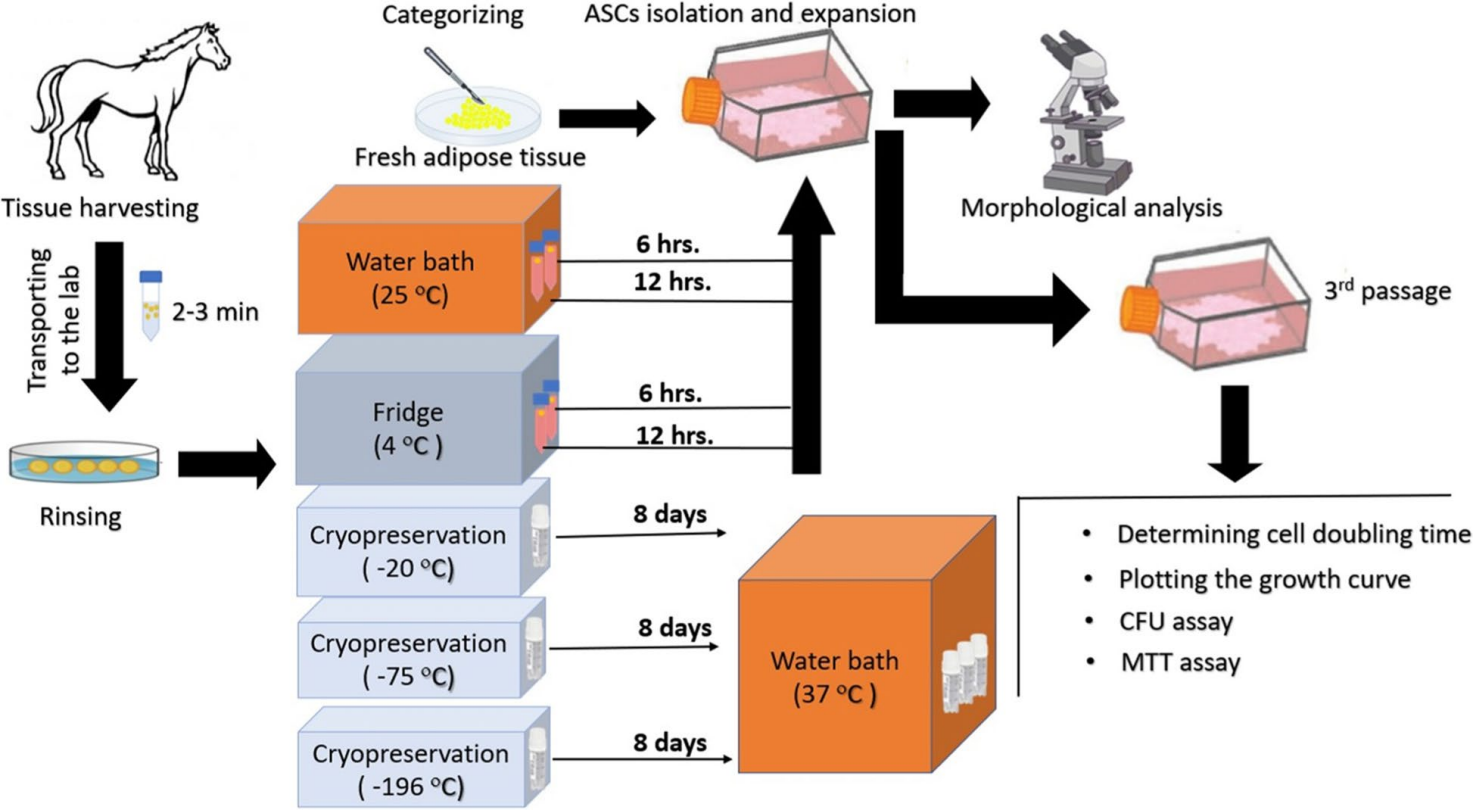
Graphic illustration of methods

Figure 7 illustrates the different stages of the study.

### Tissue harvesting

Three subcutaneous AT samples were taken from horses ranging from three to 5 years. The project underwent ethical review and was approved by the local Ethics Committee of Ferdowsi University of Mashhad (IR.UM.REC.1400.284). The care and use of experimental animals complied with local animal welfare laws, guidelines and policies.

The procedure was performed on the standing animal in a chute while sedated (Detomidine hydrochloride (Ceva, France) 0.02 mg/kg, IV and Xylazine (Alfasan, Netherland) 0.5 mg/kg, IV). The region above the dorsal gluteal muscles was clipped and anesthesia was provided by a line block (20 ml of Lidocaine Hydrochloride (Nasr, Iran) 1 ml/cm). The surgical area was prepared for aseptic surgery. A horizontal incision was made along the spinal column. Blunt dissection was made through the subcutaneous tissue down to the AT. Tissue was excised and placed in a 50 ml sterile tube filled with 40 ml phosphate buffer saline (PBS) (Biowest, France) containing amphotericin B (1 mg/ml), penicillin (100 IU/ml) and streptomycin (100 mg/ml) (Atocel, Austria) and immediately transported to the laboratory.

Skin closure was performed. After a topical antibiotic was applied, a bandage was placed on the surgical site and changed daily for 3 days. Postoperative systemic antibiotic and nonsteroidal anti-inflammatory were administered for 3 days.

### Experimental groups

The samples were washed three times with PBS supplemented with antibiotic and antifungal in the laboratory before being weighed and divided into eight groups (the amount of tissue for all experimental groups was 600 mg):

1. Control group: Fresh adipose tissue
2. Transportation via stem cell media groups:

- Two specimens of AT were placed in two conical tubes filled with 6 ml stem cell media (low glucose Dulbecco’s Modified Eagle’s Medium (DMEM) (Biosera, France)) and each was maintained at 4 ^ο^C and 25 ^ο^C for 6 hrs. (A and B, respectively).
- Two specimens of AT were placed in two conical tubes filled with 6 ml stem cell media and each was maintained at 4 ^ο^C and 25 ^ο^C for 12 hrs. (C and D, respectively).

3- Cryopreserved groups: Three specimens of AT were placed in three cryotubes and stored at − 20, − 75 and − 196 ^ο^C for 8 days E, F and G, respectively). Then, tubes were placed in a water bath and tissues were thawed at 37 ^ο^C for 15 minutes.

### Isolation and expansion of MSCs

The isolation of the MSCs procedure was based on the technique previously reported [30]. AT was washed, minced with a scalpel blade, then digested in 3.5 ml digestion media containing 0.1% collagenase type I (Bioidea, Iran) with continuous shaking at 37 ^ο^C for 60 minutes. During the last 5 min of enzymatic digestion, 0.001% DNase enzyme (Cinnage, Iran) was added to the suspension. Digested contents were filtered through a 70 μm cell strainer; an extra 3 mL of DMEM was poured through the cell strainer to collect any remaining cells from the strainer. The enzyme was neutralized with DMEM containing 10% fetal bovine serum (FBS) (Biochrom, UK). The suspension was centrifuged at 550 g for 5 minutes and the supernatant was removed. Stromal vascular fraction pellet was resuspended in DMEM. The cell concentration was calculated with a hemocytometer and a standard trypan blue assay was performed to analyze cell viability.

Finally, cells were seeded in 25 cm^2^ cell culture flasks. The flasks were incubated in a humidified chamber at 37 ^ο^C and 5% CO_2_. The cells were identified as passage (P_0_). After 3 days, unattached cells were aspirated and the media was changed every 48 hrs. Until a minimum of 70% confluency of the flasks was achieved. After being washed using PBS solution with 1% antibiotic, cells were passaged using 0.5% trypsin enzyme (Biowest, France). Cells were suspended in stem cell media, quantified and subcultured at a density of 80,000 cells/flask. Cells were also cryopreserved in DMEM with 10% DMSO (dimethyl sulfoxide) (Sigma, USA), 20% FBS and 1% antibiotic penicillin (100 IU/ml) and streptomycin (100 mg/ml) and stored at -196^ο^C for future studies. Flasks were maintained until the third passage. Then cells were utilized for subsequent evaluations.

### Morphological analysis

Cells at P_0_ were observed daily using an inverted microscope and morphological changes were recorded.

### Colony-forming assay

Cells were plated in a 35 mm cell culture dish at a density of 100 cells/dish suspended in stem cell media in three replications. Dishes were incubated in a humidified chamber at 37 ^ο^C and 5% CO_2_ for 10 days. Next, stem cell media was removed and dishes were rinsed with PBS; then 1 mL of Crystal violet 5% (Sigma, USA) was added to each plate. After 10 min of incubation at room temperature, dishes were washed with distilled water. Finally, stained colonies were fixed in 1 ml formalin 10% for an hour, formalin was aspirated and the plate was washed with distilled water and dried at room temperature. Colonies were observed under a stereomicroscope and photographed with a digital camera. The pictures were analyzed by image-processing software (image j, version 1.50 b) for colony number and surface evaluation.

### Determining cell doubling time and plotting the growth curve

Cells were seeded in a 24-well plate at a density of 10,000 cells per well and incubated at 37 ^ο^C and 5% CO_2_ in stem cell media. Adherent cells of three wells were detached by trypsinization and counted daily. The average number of three wells was calculated and recorded from days one to eight after seeding to plot the growth curve.

After the growth curve was plotted and the stationary phase of the control group was identified, cell DT was calculated for cells at this time according to the following formula:

CD: cell doubling time, Nf: cell number at the end of culture, Ni: cell number at the beginning of culture.

DT = CT/CD.

CD: cell doubling time, CT: cell culture time.

### MTT assay

The MTT (3-(4,5-dimethylthiazol-2-yl)-2,5-diphenyltetrazolium bromide) assay is used to measure cell viability. This evaluation was performed for all groups simultaneously; therefore, the frozen cells of the second passage of all groups were defrosted and seeded in a 35 mm cell culture dish. The dishes were incubated at 37 ^ο^C and 5% CO_2_ for 24 hrs.. Following this, cells were trypsinized and cultured in a 96-well plate at a density of 10,000 cells per well in three replicas and incubated in a humidified chamber at 37 ^ο^C and 5% CO_2_ in stem cell media for 24 hrs.. The culture media was aspirated, adherent cells were washed in PBS and 100 uL MTT 10X solution (Atocel, Austria) (5 mg/ml solution in PBS following continuous shaking at 4 ^ο^C for 24 hrs., filter sterilization was performed) was added. Three unseeded wells were considered as blank samples and all steps were treated like the other wells. The plate was incubated at 37 ^ο^C and 5% CO_2_ for 4 hrs.. Afterward, 100 μL Isopropanol hydrochloride was added with continuous shaking for 30 minutes at room temperature. Finally, the spectrophotometric absorbance (AB_560_) was measured using a plate reader.

### Statistical analysis

Data were statistically analyzed using Sigma Plot software. First, the normality of data was checked using the Shapiro-Wilk test (*P* < 0.05). Then, a one-way repeated measures analysis of variance was used to compare the mean in each group and between all groups. In this test, *p* values lower than 0.05 were considered statistically significant. All data are reported as mean ± SEM.

## Supplementary Information

**Additional file 1.**

## Data Availability

All data generated or analyzed during this study are included in this published article and its supplementary information files.

## Abbreviations

SC: Stem cell
AT: Adipose tissue
MSC: Mesenchymal stem cell
ASC: adipose-derived stromal/stem cells
P: Passage
CFU: Colony-forming unit
DT: Doubling time
AB560: Absorbance at 560 nm
IV: Intra-venous
PBS: Phosphate buffer saline
FBS: Fetal bovine serum
DMEM: low glucose Dulbecco’s Modified Eagle’s Medium
CD: Cell doubling time
N_f_: Cell number at the end of culture
N_i_: Cell number at the beginning of culture
CT: Cell culture time
